# Reduced subthreshold swing in a vertical tunnel FET using a low-work-function live metal strip and a low-*k* material at the drain

**DOI:** 10.3762/bjnano.15.59

**Published:** 2024-06-19

**Authors:** Kalai Selvi Kanagarajan, Dhanalakshmi Krishnan Sadhasivan

**Affiliations:** 1 Department of Electronics and Communication Engineering, Government College of Engineering, Tirunelveli, Tamil Nadu, India; 2 Kalasalingam Academy of Research and Education, Virudhunagar, Tamil Nadu, India

**Keywords:** dual low-work-function live strip (DLWLS), low-*k* dielectric spacer, low-work-function live strip (LWLS), Miller capacitance, molybdenum, subthreshold swing (SS), tunnel field-effect transistor (TFET)

## Abstract

In this research paper, a vertical tunnel field-effect transistor (TFET) structure containing a live metal strip and a material with low dielectric constant is designed, and its performance metrics are analyzed in detail. Low-*k* SiO_2_ is incorporated in the channel–drain region. A live molybdenum metal strip with low work function is placed in a high-*k* HfO_2_ layer in the source–channel region. The device is examined by the parameters *I*_off_, subthreshold swing, threshold voltage, and *I*_on_/*I*_off_ ratio. The introduction of a live metal strip in the dielectric layer closer to the source–channel interface results in a minimum subthreshold slope and a good *I*_on_/*I*_off_ ratio. The low-*k* material at the drain reduces the gate-to-drain capacitance. Both the SiO_2_ layer and the live metal strip show excellent leakage current reduction to 1.4 × 10^−17^ A/μm. The design provides a subthreshold swing of 5 mV/decade, which is an excellent improvement in TFETs, an on-current of 1.00 × 10^−5^ A/μm, an *I*_on_/*I*_off_ ratio of 7.14 × 10^11^, and a threshold voltage of 0.28 V.

## Introduction

Rapid miniaturization of electronic devices has led to an increase in leakage current. Leakage current is a big challenge in miniaturized circuits. Miniaturization, at the same time, increased the device performance and reduced the area occupied by the device. The lifetime of devices is reduced because of leakage currents [1]. Leakage increases when thin SiO_2_ is used as gate dielectric material.

The subthreshold swing (SS) is 60 mV/dec for the thermionic injection of electrons in field-effect transistors (FETs) [2]. In practical implementations, the SS is greater. Thermionic emission affects the off-to-on transition. A small subthreshold swing is needed to turn the device to an off-state sharply, once *V*_gs_ drops below the threshold voltage (*V*_T_). Tunnel field-effect transistors (TFETs) work based on band-to-band tunneling and not on conventional thermionic emission as the carrier injection mechanism [3]. Band-to-band tunneling enables TFETs to have SS < 60 mV/dec [4–7]. The gate-to-drain capacitance (*C*_gd_) effect (Miller capacitance effect) has an impact on TFET performance. Unwanted effects such as overshoot/undershoot in the inverter characteristics grow when *C*_gd_ rises [8]. The gate-to-drain capacitance increases as a result of a high-*k* material in the drain region [9]. Ambipolar leakage and Miller capacitance are two drawbacks of TFETs. The Miller capacitance can be reduced through oxide overlap and low-bandgap materials [10].

The solution to reduce gate oxide leakage are high-*k* materials. The device’s ability to keep a charge is increased by using high-*k* materials, which also aids in downsizing. HfO_2_ is compatible with a silicon substrate and possesses a high dielectric constant (ε ≈ 25), a large bandgap (5.68 eV), band offsets with silicon, a low leakage current, and a lattice parameter that is close to that of silicon with a modest lattice misfit (ca. 5%) [11].

In this paper, a published VTFET structure is taken for comparison [12]. A large tunnel area and a thin channel enhance the device metrics [13]. In contrast to the previously published model, the proposed design uses a low-*k* material in the drain region to reduce *C*_gd_. A metal strip with low work function placed at the source–channel interface causes an abrupt change in electron concentration, increasing the tunneling rate [14–18]. Molybdenum, used here as low-work-function live strip (LWLS), has a work function compatible to that of HfO_2_ [19–20]. The combination of metal strip and high-*k* material at the drain yields values of *I*_off_ = 1.40 × 10^−17^, *I*_on_/*I*_off_ ratio = 7.14 × 10^11^, a reduced subthreshold swing of 5 mV/dec, as well as lower ambipolarity and Miller capacitance.

### Impact of dielectric materials on gate source and gate–drain capacitance

The interelectrode capacitance is caused by the dielectric material of the gate. By applying a potential between the capacitor’s electrodes, the charges are polarized [9]. The capacitance of a parallel-plate capacitor is given by


[1] $$C=q/V=\varepsilon_{0}A/d,$$

where *q*, ε_0_, *A*, and *d* are the charge on the plates, the vacuum permittivity, the area of the plates, and the distance between the plates, respectively. The electric field is influenced by voltage, charge, and capacitance. The gradient of voltage that characterizes the electric field between the plates is given by


[2] E=dV/dX=V/d,

where d*V*, d*X*, *V*, and *d* stand for differential voltage, differential length, voltage, and distance between plates, respectively. Instead of SiO_2_, a high-*k* dielectric material (HfO_2_) is used to improve the capacitance by increasing the charge *q*. The increase in charge results in an increased flow of *i*(*t*) in the device. In the drain–channel region, low-*k* SiO_2_ is used. This not only reduces the drain region but also reduces the charge held in the parallel-plate capacitor (gate–drain). Hence, it reduces the undesirable *C*_gd_.

### Device structure, parameters, and simulation models

**Figure 1:**
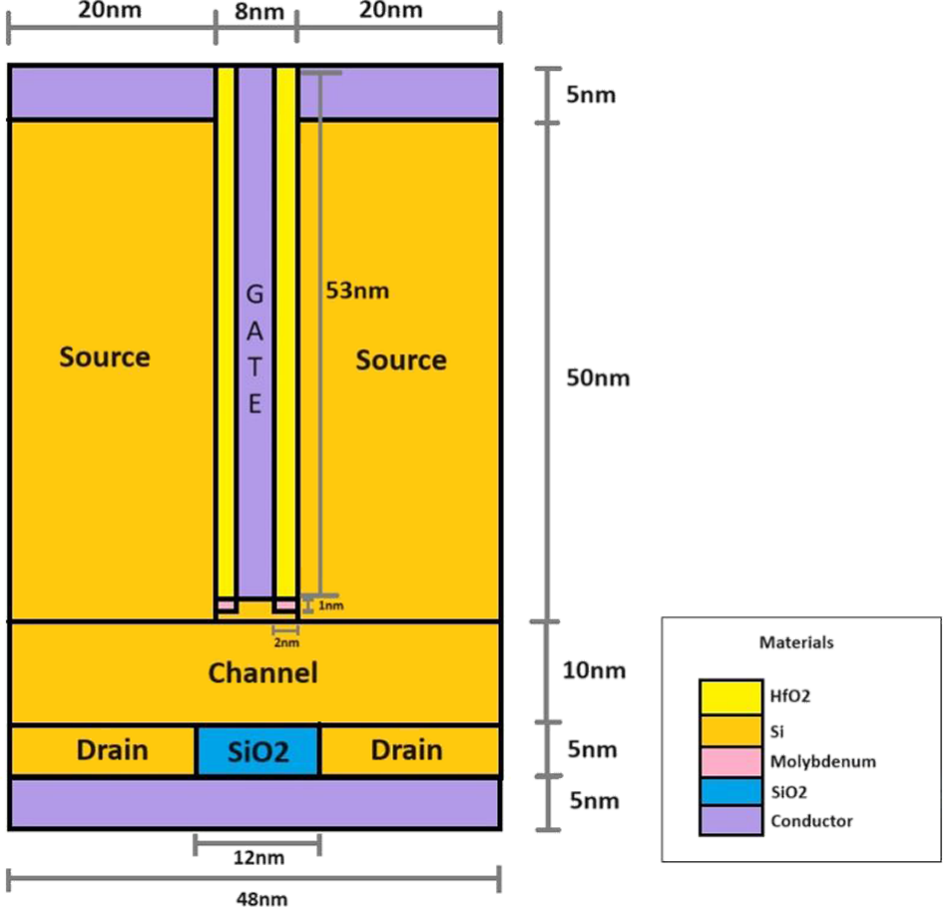
Cross-sectional schematic of the proposed VTFET with DLWLS + spacer device.

Figure 1 shows the schematic layout of the proposed vertical TFET with dual low-work-function live strip and spacer (VTFET with DLWLS + spacer), which uses molybdenum and low-*k* SiO_2_ in the drain region and HfO_2_ as a high-*k* dielectric in the source and channel regions. The gate is placed in the middle of the source. The gate length is 8 nm, the gate oxide thickness is 2 nm, and the gate height is 53 nm. The component has a symmetric dual source, measuring 50 nm in height and 20 nm on each side. The drain is 48 nm in length and 5 nm in height. The gate work function is optimized to 3.8 eV. The highly doped source (p++ type), channel (n+ type), and drain (n++ type) materials exhibit doping concentrations of 1 × 10^20^, 1 × 10^17^, and 1 × 10^19^ cm^−3^, respectively. The molybdenum live metal strip has a length of 2 nm and a height of 1 nm. The drain region is reduced by adding SiO_2_ to the middle of the drain. Molybdenum is implanted in the oxide layer (HfO_2_) near the source–channel interface and connected to gate to make it live. The work function of molybdenum is 4.53 eV. Table 1 lists the physical dimensions of the tool used for technology computer-aided design simulations.

**Table 1:** Device parameters of the VTFET with DLWLS + spacer.

source doping (NA)	1 × 10^20^ cm^−3^
drain doping (ND)	1 × 10^19^ cm^−3^
channel doping (NC)	1 × 10^17^ cm^−3^
gate height	53 nm
gate length	8 nm
source height	50 nm
source length	20 nm
drain height	5 nm
drain length	48 nm
channel height	10 nm
channel length	48 nm
gate oxide HfO_2_ thickness	2 nm
gate work function	3.8 eV
SiO_2_ thickness	5 nm
SiO_2_ length	12 nm
LWL length	2 nm
LWL thickness	1 nm
metal strip (molybdenum) work function	4.53 eV

The suggested VTFET is compared to the VTFET in [12] to demonstrate the effects of the low-work-function live strip and the low-*k* material. The device parameters used are listed in Table 1. Reference [12] used bandgap narrowing, Shockley–Read–Hall recombination, Lombardi's mobility model, and band-to-band (non-local) modeling for the simulations. The same models and experimental data have been used for the simulation of proposed structure, and the results are compared.

## Results and Discussion

### Electron concentration and electric field

**Figure 2:**
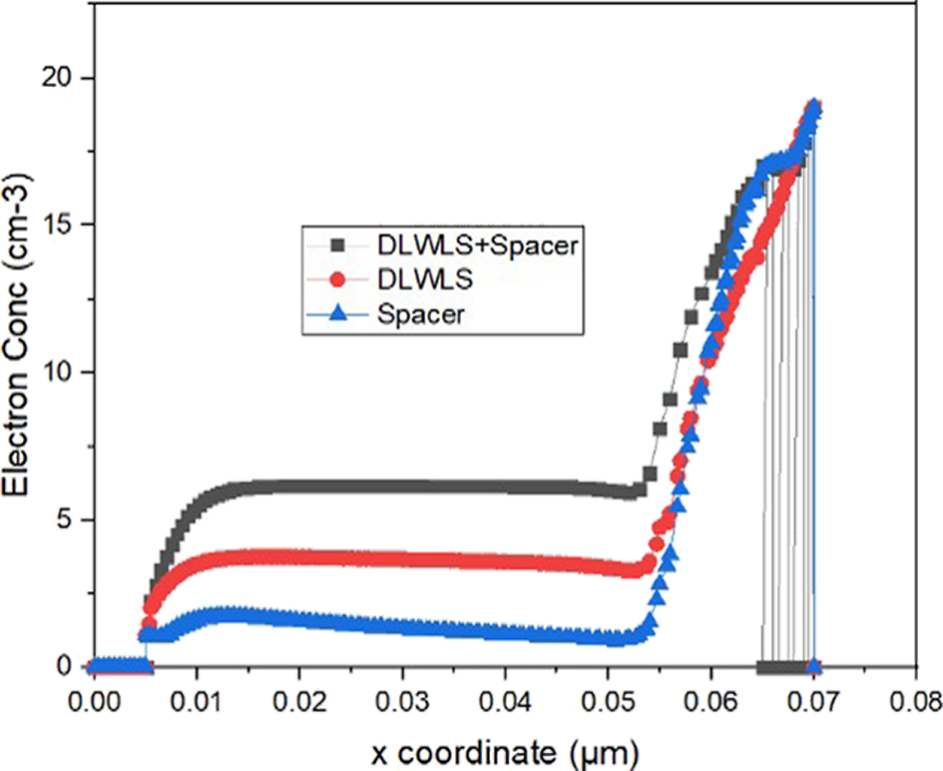
Comparison between VTFET with DLWLS + spacer, DLWLS, and spacer regarding electron concentration.

**Figure 3:**
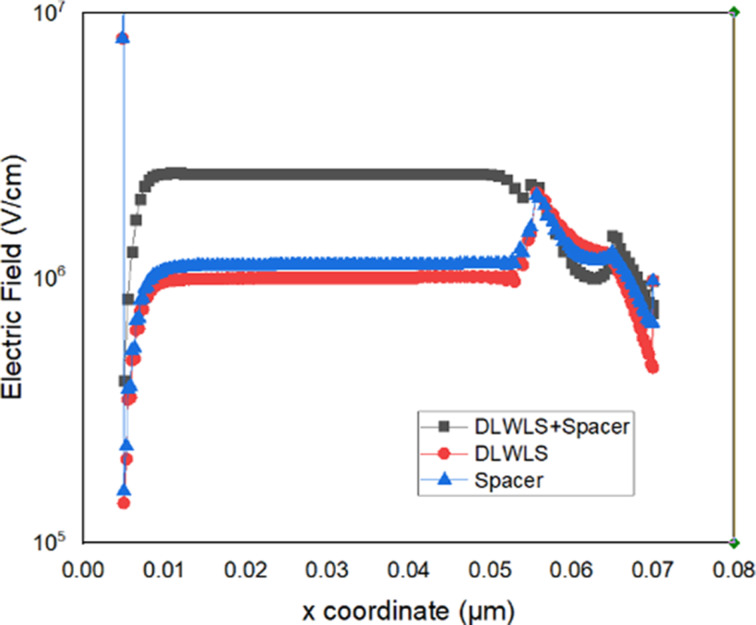
Comparison between VTFET with DLWLS + spacer, DLWLS, and spacer regarding electric field strength.

The electron concentration for a VTFET with dual low-work-function live strip and a low-*k* SiO_2_ spacer at the drain (DLWLS + spacer), a VTFET with dual low-work-function live strip (DLWLS), and a VTFET with a low-*k* SiO_2_ spacer at the drain are shown in Figure 2. Compared to the other two designs, the VTFET with DLWLS + spacer has a higher electron concentration. This is because the high-*k* dielectric gate contains a molybdenum live strip, which raises the electron concentration at the source–channel junction because of its low work function. The rise in electron concentration creates an abrupt change in the source channel. Figure 3 presents the electric field of the VTFET with DLWLS + spacer device. It exhibits a stronger electric field than the other two devices because of the increased number of charge carriers.

### Energy band

**Figure 4:**
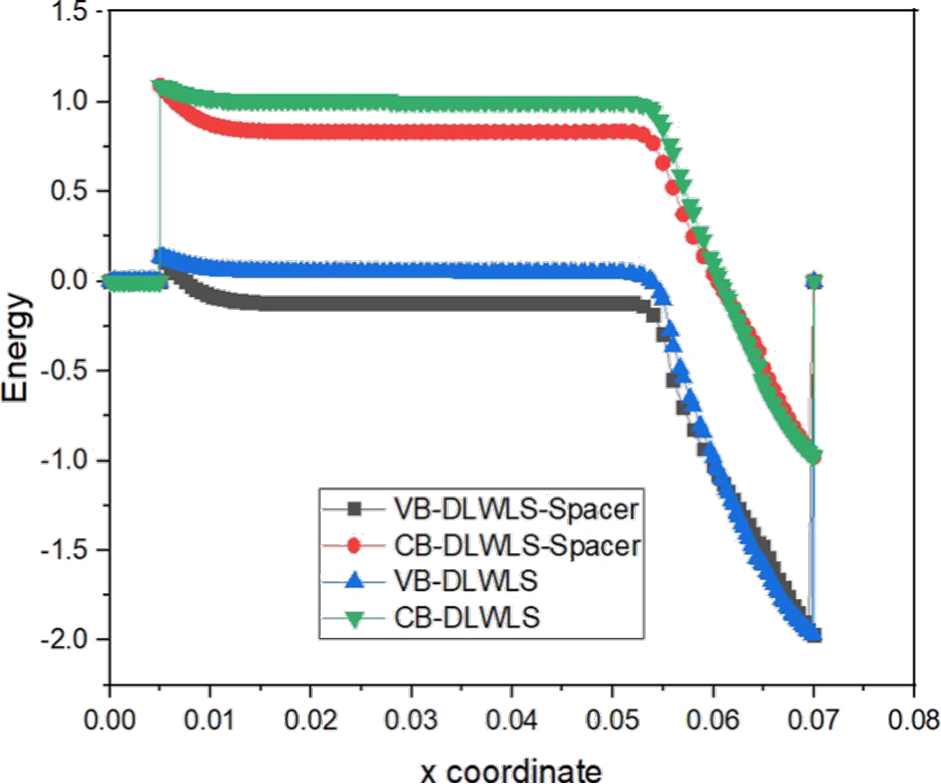
Comparison between VTFET with DLWLS + spacer, DLWLS, and spacer regarding energy band profile.

**Figure 5:**
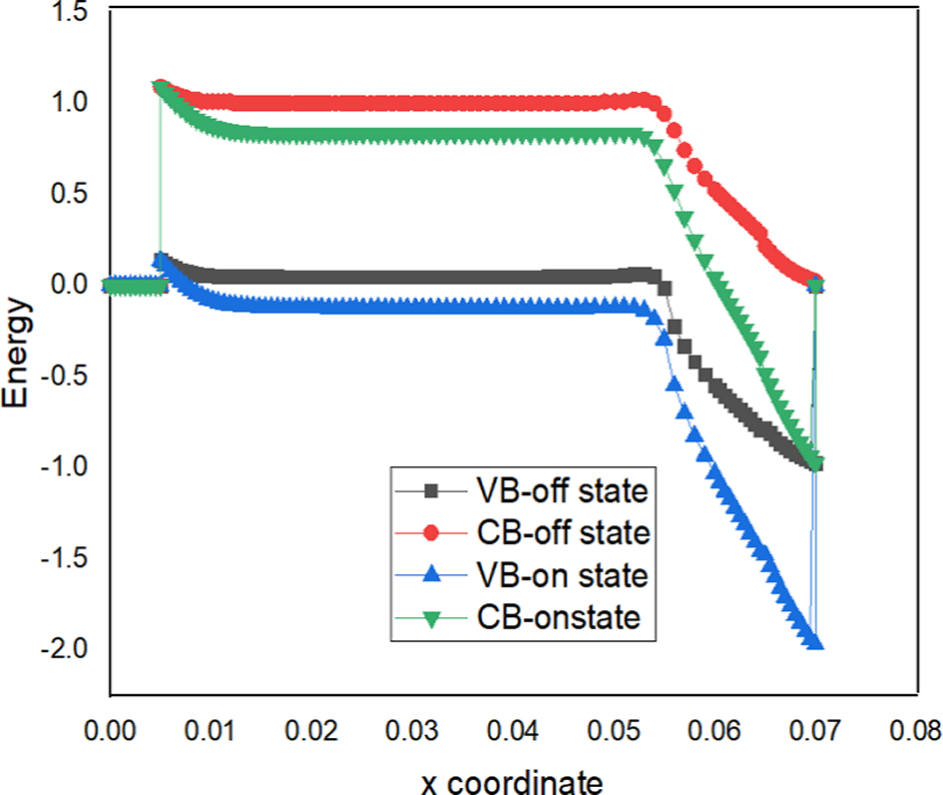
Energy band diagrams of the proposed VTFET with DLWLS + spacer in on-and off-states.

Figure 4 shows that the energy barrier is almost the same for VTFET with DLWLS and VTFET with DLWLS + spacer. The energy band diagram of the VTFET with DLWLS and spacer device is shown in Figure 5 for both the on- and the off-state. Compared to the barrier width in the on-state, the distance between valence band of the source and conduction band of the channel is greater in the off-state (*V*_gs_ = *V*_ds_ = 0). In the on-state, *V*_gs_ regulates electron motion. A reduction in barrier width enhances electron transfer in the on-state. Leakage current in the off-state is reduced by a wide tunneling barrier.

### Subthreshold swing

The gate dielectric material and the geometry of the transistor help in reducing the subthreshold swing. The subthreshold swing is 5 mV/dec for VTFET with DLWLS + spacer, 25 mV/dec for DLWLS, and 30 mV/dec for the design with only the spacer. The subthreshold swing of a TFET depends on the high-*k* gate dielectric and a thin body to assure that the gate field directly modulates the channel. Maximizing the derivative of the junction electric field on the gate–source voltage [4] is another method for reducing the subthreshold voltage swing. *V*_eff_ is the tunnel–junction bias, ξ is the electric field, *a*, *b* are coefficients based on the junction's material characteristics and the device’s cross-sectional area:


[3] $$\text{SS}=\log{\left[\frac{1}{V_{\text{eff}}}\frac{\text{d}V_{\text{eff}}}{\text{d}V_{\text{gs}}}+\frac{\xi +b}{\xi^{2}}\frac{\text{d}\xi }{dV_{\text{gs}}}\right]}^{-1}.$$

### Comparison of VTFET with VTFET (DLWLS and Spacer)

**Figure 6:**
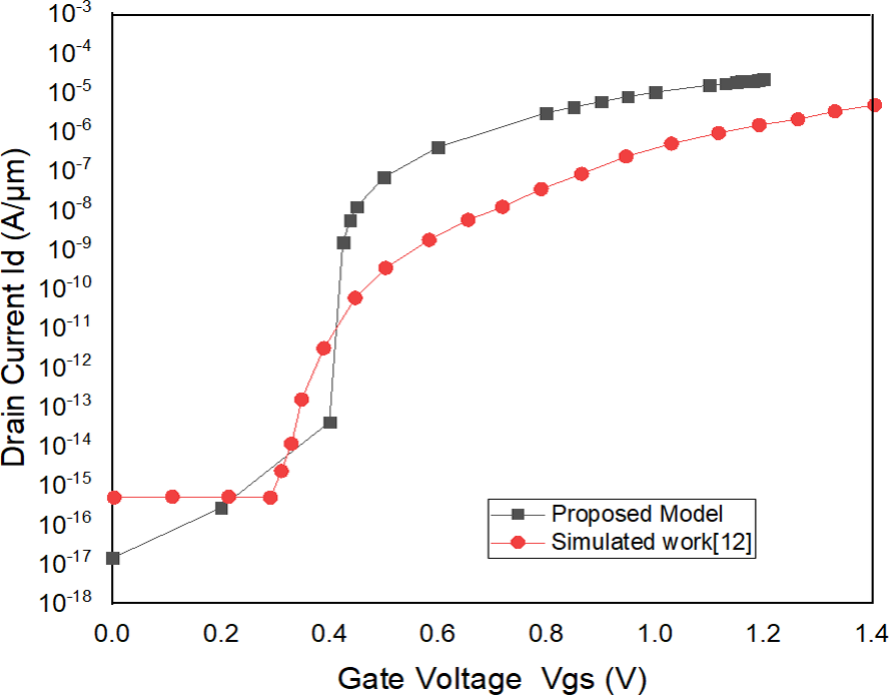
*^I^*_d_^vs ^*^V^*_gs_^of the VTFET with DLWLS + spacer and the model in^ [12].

**Figure 7:**
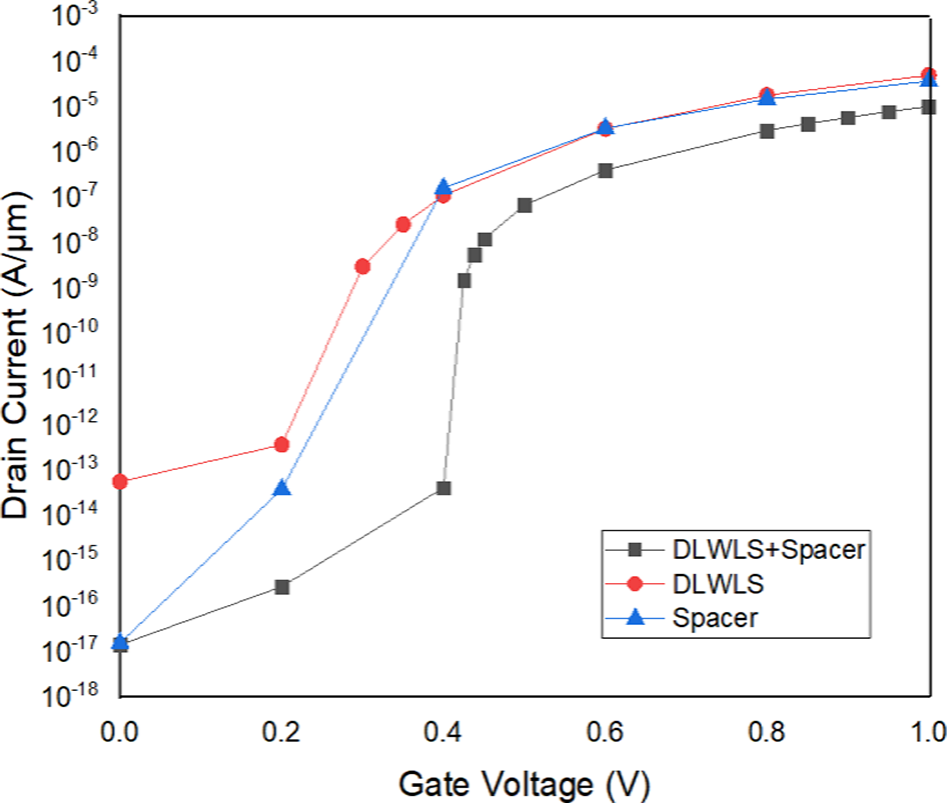
Comparison between transfer characteristics of VTFET with DLWLS + spacer, DLWLS, and spacer.

The proposed model VTFET with DLWLS + spacer is compared with the previously published work [12]. The *I*_d_ vs *V*_gs_ curve of the model proposed here is better in terms of leakage current as illustrated in Figure 6. The off-current of the proposed model is 1.40 × 10^−17^ A/μm, while that of the model in [12] is 2.96 × 10^−13^ A/μm. The threshold voltage is 0.28 V. The on-state current is 1.00 × 10^−5^ A/μm. The transfer characteristics of the three devices are presented in Figure 7. The leakage current is minimal in the proposed model.

### Miller capacitance and leakage

**Figure 8:**
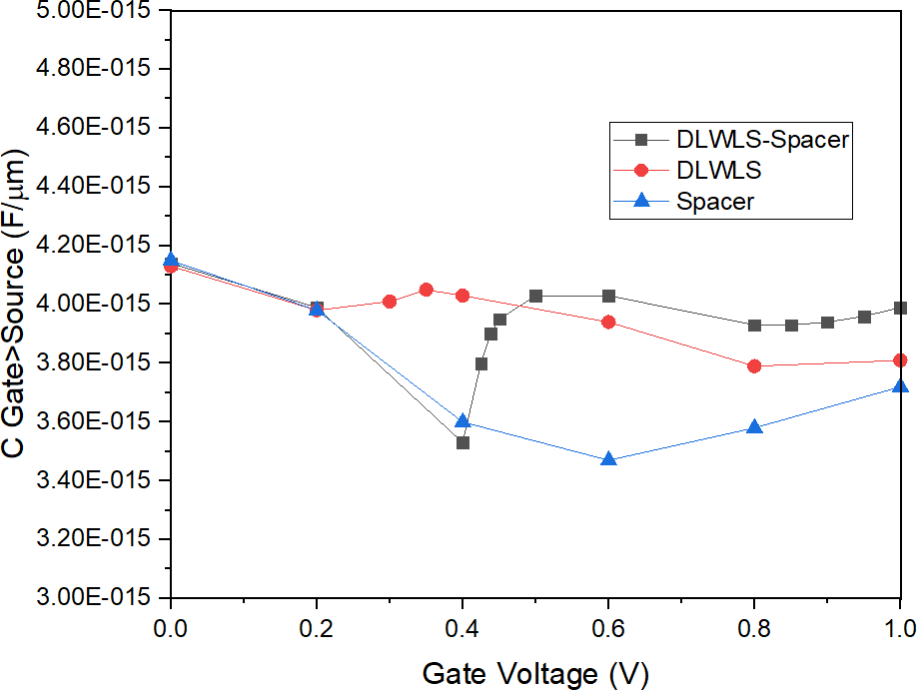
Capacitance (*C*_gs_)–voltage characteristics of the three models.

**Figure 9:**
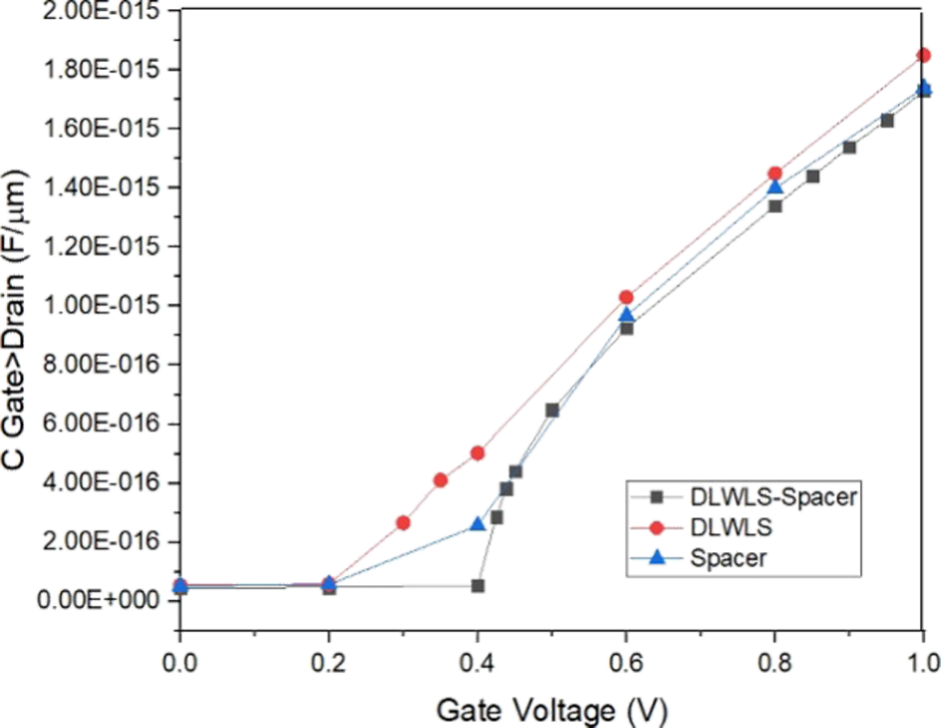
Capacitance (*C*_gd_)–voltage characteristics of the three models.

The high dielectric constant of HfO_2_ increases the capacitance at the source–channel junction. This increases *C*_gs_; however, employing low-*k* SiO_2_ at the drain decreases the Miller capacitance, which is desirable for FETs [21]. Figure 8 and Figure 9 show the increase in *C*_gs_ and the decrease in *C*_gd_, respectively, for the proposed model. The proposed structure has been simulated with and without dielectric at the drain. The leakage current in presence of the dielectric is 1.40 × 10^−17^ A/μm, which is small compared to the value without dielectric, which is 5.47 × 10^−14^ A/μm. Low-*k* SiO_2_ reduces the scattering and the charge concentration in the drain region, reduces unwanted capacitance, and acts as a collecting region. The *I*_on_/*I*_off_ value obtained for the proposed structure is 7.14 × 10^11^. The values for existing structures are 10^8^ to 10^9^. The proposed structure has been designed to have a moderate *V*_T_ of 0.28 V since FETs with low threshold voltage are used in high-speed designs, but at the cost of higher leakage power consumption. The device previously described in the literature has a low threshold voltage of 0.15 V and a higher off-current than the design proposed here. Hence, the proposed design can be employed in low-power applications.

## Conclusion

In this proposed work, a high-*k* gate oxide with molybdenum metal is designed along with a low-*k* material at the drain. FETs are playing a vital role in the IC industry, and low sub-threshold swing and reduced Miller capacitance with low off-state current are required for power-efficient low-leakage memory systems. Hence, a VTFET with a low-work-function live strip and a spacer has been designed for maximum performance by limiting the leakage current. The performance of the proposed VTFET with DLWLS + spacer is presented and compared with standalone DLWLS or spacer structures. Miller capacitance, subthreshold swing, and leakage of the proposed device are improved compared to other architectures because of the low-work-function metal and the low-*k* material at the drain. The suggested model has been implemented with the help of the Atlas Silvaco tool.

## Data Availability

Additional research data is not shared.
